# Rapid detection of carbapenemase production in *Aeromonas* using phenotypic tests based on colorimetric microtube assay

**DOI:** 10.1128/jcm.01104-24

**Published:** 2024-12-05

**Authors:** Hui Liu, Lijun Zhang, Min Jiang, Yuhong Zhang, Bin Sun

**Affiliations:** 1 Department of Blood Transfusion, The First Affiliated Hospital of Chongqing Medical University 117972 , Chongqing, China; 2 Department of Laboratory Medicine, The Second Affiliated Hospital of Chongqing Medical University 585250 , Chongqing, China; Johns Hopkins University, Baltimore, Maryland, USA

**Keywords:** *Aeromonas*, carbapenemase, phenotypic test, c-CNPt and ec-CNPt

## Abstract

Antibiotic resistance, particularly carbapenem resistance, poses a significant global health threat due to the limited availability of effective antibiotics. Carbapenem-resistant *Aeromonas* are increasingly recognized for their role in various infections, necessitating rapid and accurate detection methods. This study aimed to evaluate several phenotypic tests, including the Carba NP test (CNPt), Carba NP-direct test (CNPd), and Blue-Carba test (BCT), for their effectiveness in rapidly detecting carbapenemase production in *Aeromonas*. These tests target both the chromosomally encoded CphA metallo-β-lactamase (MBL) and acquired carbapenemases. Additionally, a modified phenotypic test called the Colony-Carba NP test (c-CNPt) was introduced to enhance sensitivity and specificity. A retrospective analysis was conducted on 131 clinically conserved *Aeromonas* strains harboring identified carbapenem resistance genes, using CNPt, CNPd, BCT, and the newly developed c-CNPt and EDTA-Colony-Carba NP test (ec-CNPt). The stability of c-CNPt reagents stored at −80°C was also assessed. Additionally, a prospective study conducted from July 2021 to November 2023 evaluated 152 *Aeromonas* isolates to determine the clinical applicability of these tests. Our results demonstrated that CNPd and BCT achieved 100% sensitivity and specificity, surpassing the traditional CNPt, which showed only 63.6% sensitivity for *Aeromonas* strains. The c-CNPt also showed 100% sensitivity and specificity, with the ec-CNPt effectively differentiating between MBL and serine carbapenemase types. Stability tests confirmed that c-CNPt reagents could be stored at −80℃ for up to 1 year without performance degradation. These findings highlight the practicality and reliability of these phenotypic tests for routine laboratory use, providing a rapid and cost-effective method for detecting carbapenemase production.

The rapid detection of carbapenemase production in *Aeromonas* is of paramount importance due to the significant clinical and public health implications associated with antibiotic resistance. The development and validation of rapid phenotypic tests such as the Colony-Carba NP test (c-CNPt) and the EDTA-Colony-Carba NP test (ec-CNPt) are crucial advancements in the field. These tests offer a highly sensitive and specific method for detecting carbapenemase production in *Aeromonas*, including the differentiation between metallo-β-lactamase and serine carbapenemases. The c-CNPt and ec-CNPt are cost-effective, easy to perform, and provide rapid results, making them suitable for routine clinical use. Additionally, the stability of the reagents ensures their practicality for long-term application in various healthcare settings. Implementing these phenotypic tests in clinical laboratories can significantly enhance the early detection and appropriate treatment of carbapenem-resistant *Aeromonas* infections.

## INTRODUCTION

Antibiotic resistance, especially carbapenem resistance, has emerged as a grave global health threat due to the limited availability of effective antibiotics for pathogen treatment. Infections caused by carbapenem-resistant Gram-negative bacteria are associated with elevated financial burdens, prolonged hospitalizations, and increased morbidity and mortality risks compared to those caused by susceptible strains (1). Carbapenem-resistant *Enterobacterales* (CRE), *Acinetobacter baumannii* (CRAB), and *Pseudomonas aeruginosa* (CRPA) represent the most critical priority group of multidrug-resistant bacteria listed by the World Health Organization to guide research, discovery, and development of new antibiotics (2). Their primary mechanism of resistance involves the production of carbapenemases that hydrolyze carbapenems. The genes encoding carbapenemases are typically located on plasmids, which exhibit high transmissibility (1, 3). While the focus primarily lies on CRE, CRAB, and CRPA pathogens, it is imperative not to overlook *Aeromonas*.

*Aeromonas* are ubiquitously distributed in the environment and can cause gastroenteritis and extra-intestinal infections (4, 5). Effective antibiotic treatment is crucial for symptom relief, prognosis improvement, and mortality reduction. CphA, a distinctive chromosomally encoded metallo-β-lactamase (MBL) found exclusively in *Aeromonas*, hydrolyzes only carbapenems (6, 7). Previous studies have shown that up to 60–77% of *Aeromonas* contain the CphA MBL. However, some CphA-positive strains may test susceptible to carbapenems in routine antimicrobial susceptibility testing (AST), as the enzyme’s activity can sometimes be low, leading to variable detection of carbapenem resistance (6–8). Moreover, CphA MBL is an inducible carbapenemase that can undergo mutations and conformational changes under carbapenem selective pressure *in vitro*, resulting in enhanced hydrolysis ability (9, 10). The empirical use of carbapenem antibiotics poses a risk factor for developing carbapenem-resistant *Aeromonas*. In addition to CphA MBL, other mobile genetic elements carrying diverse types of carbapenemases, such as KPC-2, NDM-1, OXA-181, and GES-24, have also emerged among *Aeromonas* isolates (8, 11, 12). Horizontal gene transfer and clonal dissemination play a significant role in the development of resistance against carbapenems among *Aeromonas* (13, 14). Therefore, early detection of carbapenem-resistant *Aeromonas* strains is critical.

Our previous research demonstrated the effectiveness of modified carbapenem inactivation method (mCIM) in detecting multiple carbapenemases in *Aeromonas*, showing high specificity and sensitivity (8). However, the long detection time required by this method is not suitable for rapid identification of carbapenem-resistant *Aeromonas*. The Carba NP test (CNPt), recommended by the Clinical and Laboratory Standards Institute (CLSI) for *Enterobacterales* and *P. aeruginosa*, is proven to be an effective method for detecting carbapenemases within 2 h (15). It can also detect CphA activity in *Aeromonas* (7). Building upon the CNPt, several related tests have been developed, including the Carba NP-direct test (CNPd) and the Blue-Carba test (BCT). The CNPd eliminates the need for a lysis process and reduces testing costs by using bacterial colonies directly (16). The BCT utilizes bromothymol blue as an indicator, with positive results appearing as yellow or green, and has also shown good detection performance (17). However, there are no reports on the application of these two methods in detecting carbapenemases in *Aeromonas*. Therefore, it is of great significance to find a more accurate and rapid method for detecting carbapenem-resistant *Aeromonas*.

This study retrospectively evaluated the application of CNPt, CNPd, and BCT in detecting carbapenemases in *Aeromonas* using 131 strains with identified carbapenem-resistance genes. Combining these tests, we designed a simpler phenotype detection method called the Colony-Carba NP test (c-CNPt), which, compared to CNPt, directly uses bacterial colonies without adding additional reagents, demonstrating extremely high sensitivity and specificity. We also validated the feasibility of the EDTA-Colony-Carba NP test (ec-CNPt) in differentiating carbapenemase types. Additionally, we assessed the stability of reagents stored at −80℃ for one year to reduce the frequency of reagent preparation. Furthermore, a prospective study of *Aeromonas* isolated from July 2021 to November 2023 was conducted to evaluate the clinical application of these tests. Our findings hold significant implications for effectively treating carbapenem-resistant *Aeromonas* in clinical institutions.

## MATERIALS AND METHODS

### Bacterial isolates

A total of 131 clinically conserved *Aeromonas* strains harboring identified carbapenem resistance genes, including *bla*KPC, *bla*NDM, and *bla*CphA, were retrospectively analyzed to evaluate the effectiveness of CNPt and modified tests for rapid detection of carbapenemases. The presence of these resistance genes was determined through PCR amplification and subsequently confirmed by Sanger sequencing. Among the 131 strains of *Aeromonas*, 68 contained *bla*CphA, 6 contained *bla*KPC-2, 2 harbored *bla*NDM-1, and 3 carried both *bla*KPC-2 and *bla*CphA. The isolates, stored at −80°C, were subcultured twice at 35°C for 18–24 h on blood agar plates (Oxoid Ltd, Basingstoke, UK) before testing. The microorganisms were identified using the VITEK2 system (bioMerieux, Hazelwood, MO, USA) and confirmed by Clin-ToF II MS (Bioyong Technologies, Beijing, China). *Klebsiella pneumoniae* ATCC 1705 and ATCC 1706 served as positive and negative quality controls, respectively, for all phenotypic tests performed in this study. Quality controls were tested with every batch of samples to ensure the accuracy and consistency of the results.

### Carba NP test

The CNPt was performed according to the CLSI M100 with certain modifications (15). Solution A consisted of 0.05% phenol red and 0.1 mmol/L ZnSO4 (Sangon Biotech, Shanghai, China). Solution B was prepared by mixing solution A with 12 mg/mL imipenem-cilastatin (equivalent to 6 mg/mL of imipenem) (Tienam 500, Merck Sharp & Dohme, USA), resulting in a final pH of 7.8. A full loop (approximately 10 µL) of bacteria from a blood agar plate was emulsified in 100 µL of bacterial protein extraction reagent II (B-PER II, Thermo Scientific, Rockford, IL, USA), vortexed for 5 s, and then added to 100 µL of either solution A or solution B, respectively. The tubes were incubated at 35°C for up to 2 h, with a positive result of CNPt interpreted by a color change from red to light orange, yellow, or dark yellow.

### CNPt-direct test

The CNPd was performed using colonies directly, rather than bacterial extracts (16). 0.1% vol/vol Triton X-100 (Sangon Biotech, Shanghai, China) was added to both solutions A and B before adjusting the pH. Subsequently, 1 µL of colonies was emulsified in 100 µL of either solution A or B, with results observed within 2 h. The color transformation of solution B from red to light orange, yellow, or dark yellow indicated the presence of carbapenemase.

### Blue-Carba test

The BCT was performed following the protocol as described previously with minor modifications (17). Bromothymol blue (Sangon Biotech, Shanghai, China) served as an indicator in the BCT assay. Solution A contained 0.04% bromothymol blue and 0.1 mmol/L ZnSO4. Solution B was prepared by adding 6 mg/mL imipenem-cilastatin (equivalent to 3 mg/mL imipenem) to solution A and adjusting the pH to 7.0. Subsequently, 5 µL of colonies was mixed with 100 µL of either solution A or B, followed by incubation at 35°C with continuous monitoring for 2 h. Carbapenemase activity was confirmed when solution B, compared to solution A, exhibited distinct color changes: (i) yellow to blue, (ii) yellow to green, or (iii) green to blue.

### Colony-Carba NP test and EDTA-Colony-Carba NP test

**Fig 1:**
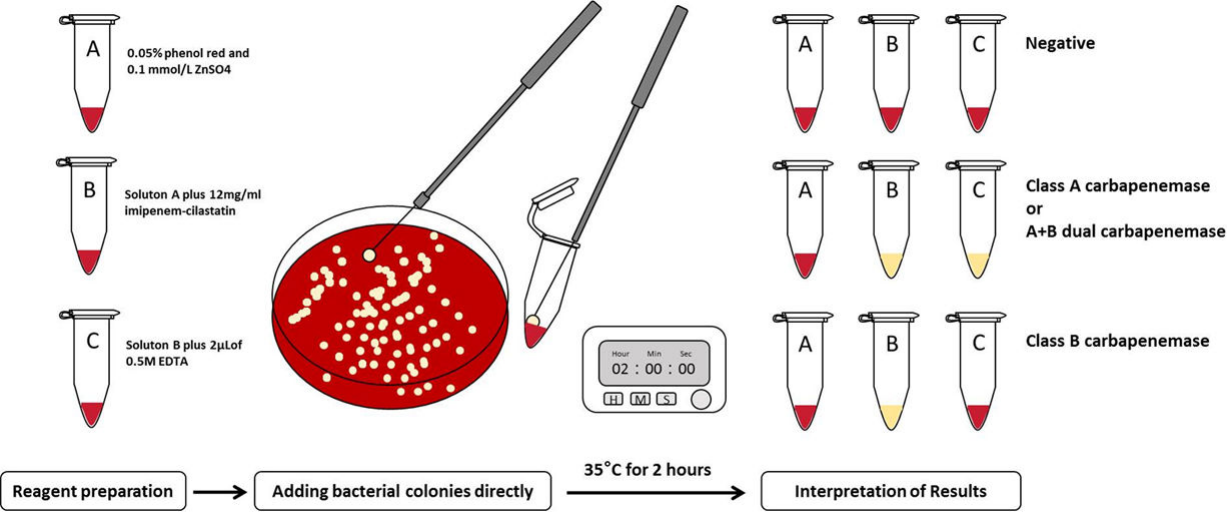
Flowchart of c-CNPt and ec-CNPt.

We modified the CNPt by using bacterial colonies directly instead of bacterial extracts, naming it c-CNPt and ec-CNPt. Solutions A and B for c-CNPt remained the same as those used in the original CNPt. For ec-CNPt, solution C was prepared by adding 2 µL of 0.5 M EDTA to solution B. Then, 5 µL of bacterial colonies was suspended directly in 100 µL of solution A, B, or C, respectively, and incubated at 35°C for 2 h. Carbapenemase activity was confirmed when the color of solution B changed from red to light orange, yellow, or dark yellow. Carbapenemase type was then determined by comparing the color change of solution C with solution B as follows: (i) red versus yellow for MBL and (ii) yellow versus yellow for serine carbapenemase or dual carbapenemase. The flowchart of the specific operation is shown in Fig. 1.

### Reagent stability test

The reagents for the c-CNPt test were stored in multiple aliquots at −80°C, with each aliquot containing 100 µL of solution A or B to minimize freeze-thaw cycles and maintain reagent stability. Stability tests validated the effectiveness of the reagents over a 12-month period without significant degradation. Prior to each experiment, solutions A and B should be equilibrated to room temperature. Subsequently, 2 µL of 0.5 M EDTA was added to a separate tube containing solution B to prepare solution C. A total of 131 *Aeromonas* strains were divided into six groups, each consisting of 21 or 22 isolates. Groups 1–6 were tested at the end of their corresponding month, and this schedule was repeated for Months 7 through 12 (e.g., Group 1 was tested at the end of Months 1 and 7). The positive controls for each trial included *K. pneumoniae*-producing KPC and *Escherichia coli*-producing NDM, both of which were preserved in our laboratory and sequence-verified to ensure accuracy and reproducibility.

### Prospective study

For AST, We employed the VITEK2 system using GN67 and XN04 cards, following the manufacturer’s protocols and interpreting results in accordance with the CLSI M45 guidelines (18). Phenotypic tests, including CNPt, CNPd, BCT, c-CNPt, and ec-CNPt, were routinely performed on 152 identified *Aeromonas* isolates from July 2021 to November 2023 as a supplement to AST. Bacterial genomic DNA was extracted using a Spin Column Bacterial Genomic DNA Isolation Kit (Sangon Biotech, Shanghai, China). Subsequently, the presence of carbapenemase-encoding genes was determined by PCR amplification, targeting the following genes: *bla*CphA, *bla*KPC, *bla*NDM, *bla*IMP, *bla*IMI, *bla*GES, *bla*OXA-48, *bla*VIM, *bla*SME, *bla*GIM, and *bla*SIM. The PCR thermal cycling procedures and primers used in this study were described previously (8). All positive amplicons were further confirmed through commercial direct sequencing (Sangon Biotech, Shanghai, China). The genetic results ultimately served as the gold standard for comparison with the results of c-CNPt and ec-CNPt.

### Statistical analysis

Phenotypic tests were conducted in duplicate with results independently interpreted by three technicians. Statistical analyses were conducted using SPSS software version 22.0 (SPSS Inc., Chicago, IL, USA). Descriptive statistics were presented as numbers and percentages. Cochran’s *Q* test, along with McNemar’s test, was employed to assess the differences in resistance rates among various carbapenem antibiotics. The 95% confidence intervals (CIs) for sensitivity, specificity, and accuracy were calculated using gene results as the gold standard. In this study, indeterminate results of phenotypic tests were classified as false negatives.

## RESULTS

### Phenotypic test results in retrospective analysis

**Fig 2:**
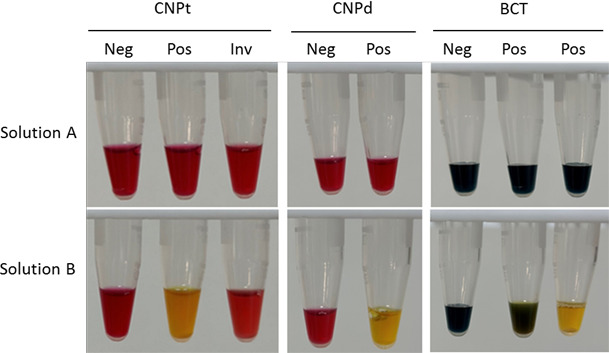
Representative results of CNPt, CNPd, and BCT. Photographs were taken after a 2-h incubation using an iPhone 14 Pro Max. In the CNPt, a color change in solution B from red to yellow indicates a positive result, whereas a change from red to red-orange is considered invalid. In CNPd, a positive result is indicated by a color change in solution B from red to yellow. In BCT, a positive result is indicated by a color change in solution B from blue to green or yellow. Neg, negative; Pos, positive; Inv, invalid.

*Aeromonas* isolates carrying KPC-2 (nine isolates) and NDM-1 (two isolates) tested positive for carbapenemase using CNPt. Of the 68 strains with CphA, only 37 tested positive for CNPt, 23 had invalid results, and 8 were negative. All 52 gene-negative bacteria tested negative for CNPt (Table 1; Fig. 2). Both CNPd and BCT revealed that all 79 gene-positive strains showed positive results for carbapenemase. The detection time for KPC-2 and NDM-1 positive strains was shorter than that for CphA-positive strains. Furthermore, 52 isolates lacking the target gene were confirmed as carbapenemase-negative based on results from both the CNPd and BCT assays (Table 1; Fig. 2).

**TABLE 1:** Results of phenotypic tests in retrospective analysis

Carbapenemase genes (*n*)	CNPt (*n*, %)		BCT (*n*, %)		CNPd (*n*, %)		c-CNPt (*n*, %)		ec-CNPt (*n*, %)	
	+	−	+	−	+	−	+	−	Serinase	MBL
Positive (79)	48 (60.8)	31 (39.2)	79 (100)	0 (0)	79 (100)	0 (0)	79 (100)	0 (0)	9 (11.4)	70 (88.6)
CphA (68)	37 (54.4)	31 (45.6)	68 (100)	0 (0)	68 (100)	0 (0)	68 (100)	0 (0)	0 (0)	68 (100)
KPC-2 (6)	6 (100)	0 (0)	6 (100)	0 (0)	6 (100)	0 (0)	6 (100)	0 (0)	6 (100)	0 (0)
NDM-1 (2)	2 (100)	0 (0)	2 (100)	0 (0)	2 (100)	0 (0)	2 (100)	0 (0)	0 (0)	2 (100)
KPC-2 + CphA (3)	3 (100)	0 (0)	3 (100)	0 (0)	3 (100)	0 (0)	3 (100)	0 (0)	3 (100)	0 (0)
Negative (52)	0 (0)	52 (100)	0 (0)	52 (100)	0 (0)	52 (100)	0 (0)	52 (100)	/	/

**Fig 3:**
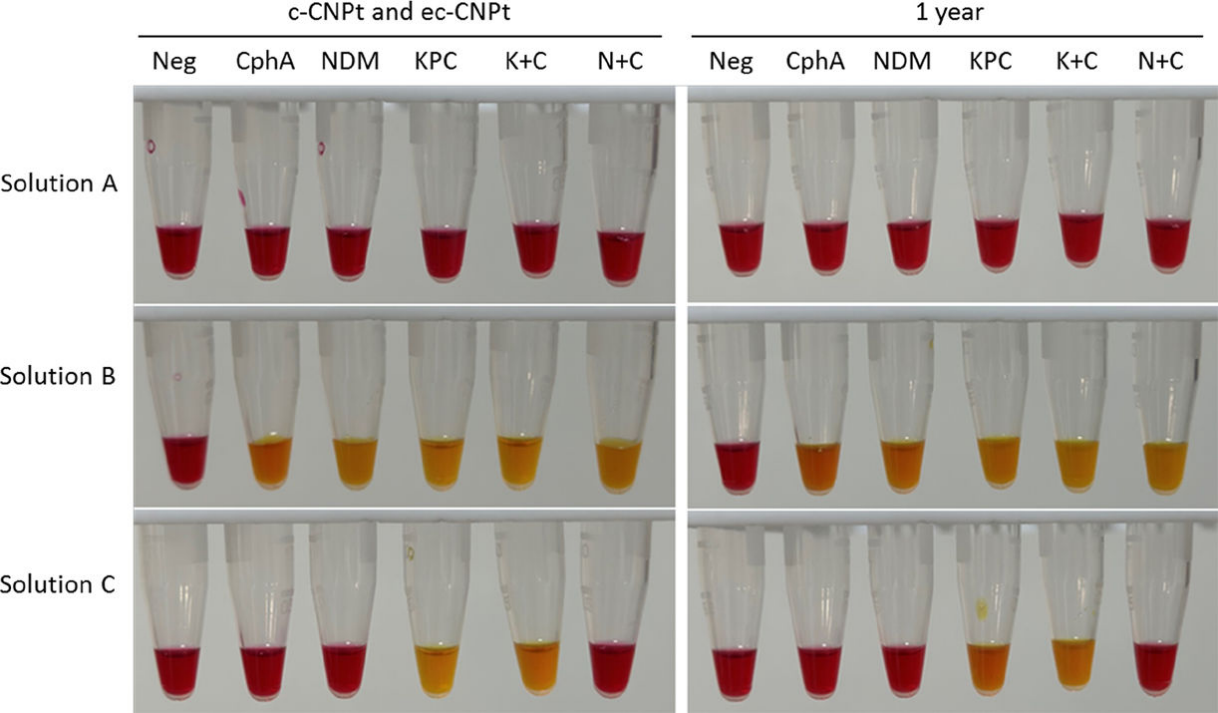
Representative results of c-CNPt and ec-CNPt. Photographs were taken after a 2-h incubation using an iPhone 14 Pro Max. In c-CNPt, a color change in solution B from red to yellow indicates a positive result for carbapenemase production. In ec-CNPt, a red color in solution C indicates the production of class B carbapenemase, whereas a yellow color indicates the production of class A carbapenemase or a combination of class A + B dual carbapenemases. The results obtained with reagents stored at −80°C for 1 year were consistent with those obtained using freshly prepared reagents. Neg, negative; K+C, KPC + CphA; N+C, NDM + CphA.

Out of 131 isolates, 79 revealed positive results for carbapenemase, while 52 showed negative results using c-CNPt, aligning perfectly with the gene testing findings. The ec-CNPt results revealed that among the 68 CphA-positive strains and 2 NDM-1-positive strains, the color of solution C remained unaltered upon the addition of EDTA, indicating the presence of MBL. Conversely, for six strains carrying KPC-2 and three strains expressing both KPC-2 and CphA, solution C turned yellow, suggesting a serine carbapenemase phenotype (Table 1; Fig. 3).

### Stability test results of c-CNPt reagent

We monitored the stability of the c-CNPt reagent over a 1-year period. After equilibrating to room temperature, the reagents showed no significant color change before testing. The color changes observed in solutions A, B, and C were consistent with the initial detection results using freshly prepared reagents for 79 positive strains and 52 negative strains, achieving a 100% conformity rate over the 1-year period. The initial and final results are shown in Fig. 3.

### Prospective trial results

**Fig 4:**
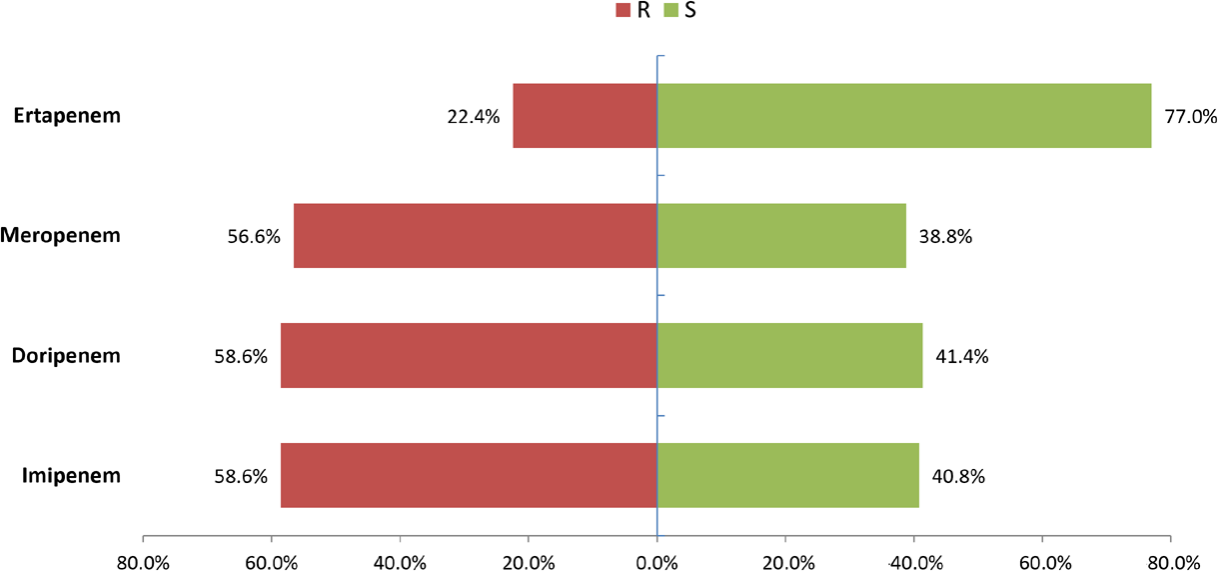
Antibiotic susceptibility testing results of carbapenems using the VITEK2 system in the prospective study. McNemar’s test showed that the resistance rate of ertapenem was significantly lower than that of imipenem, meropenem, and doripenem (all *P* < 0.001). Compared to PCR genotyping, which identified resistance genes in 63.8% of isolates, the VITEK2 system may overlook certain CphA-positive strains.

Among the 152 *Aeromonas* strains, imipenem and doripenem exhibited the highest resistance rates at 58.6%, followed by meropenem at 56.6%. In contrast, ertapenem had a significantly lower resistance rate of 22.4% compared to the other carbapenems, with statistical significance noted for both comparisons (both *P* < 0.001) (Fig. 4). PCR detection revealed that 97 strains, accounting for 63.8%, carried carbapenem resistance genes. Among these, 82 strains were CphA-positive, 1 strain was KPC-positive, 13 strains were NDM-positive, and 1 strain contained both NDM and CphA. Phenotypic tests indicated that all 97 strains were positive for CNPd, BCT, and c-CNPt. However, only 64 strains were positive for CNPt, while the remaining 33 CphA-positive strains showed invalid or negative results (Table 2). Additionally, ec-CNPt could effectively distinguish between MBL and serine carbapenemase (Table 2).

**TABLE 2:** Genes and phenotypic tests results in prospective study

Carbapenemase genes (n)	CNPt (*n*, %)		BCT (*n*, %)		CNPd (*n*, %)		c-CNPt (*n*, %)		ec-CNPt (*n*, %)	
	+	−	+	−	+	−	+	−	Serinase	MBL
Positive (97)	64 (66.0)	33 (34.0)	97 (100)	0 (0)	97 (100)	0 (0)	97 (100)	0 (0)	1 (1.0)	96 (99.0)
CphA (82)	49 (59.8)	33 (40.2)	82 (100)	0 (0)	82 (100)	0 (0)	82 (100)	0 (0)	0 (0)	82 (100)
KPC-2 (1)	1 (100)	0 (0)	1 (100)	0 (0)	1 (100)	0 (0)	1 (100)	0 (0)	1 (100)	0 (0)
NDM-1 (13)	13 (100)	0 (0)	13 (100)	0 (0)	13 (100)	0 (0)	13 (100)	0 (0)	0 (0)	13 (100)
NDM-1 + CphA (1)	1 (100)	0 (0)	1 (100)	0 (0)	1 (100)	0 (0)	1 (100)	0 (0)	0 (0)	1 (100)
Negative (55)	0 (0)	33 (34.0)	0 (0)	55 (100)	0 (0)	55 (100)	0 (0)	55 (100)	/	/

### Sensitivity and specificity of phenotypic tests

Analysis of phenotypic tests on 283 strains revealed that CNPt exhibited a sensitivity of 63.6% (95% CI: 56.0–70.6%) and a specificity of 100% (95% CI: 95.7–100%). However, false negative outcomes were observed in certain CphA-producing strains, resulting in an overall concordance rate of 77.1% (95% CI: 72.2–81.9%) (Table 3). Notably, while not all false negatives were susceptible to carbapenems, none demonstrated complete resistance to all four carbapenems. In contrast, CNPd, BCT, and c-CNPt demonstrated a sensitivity and specificity of 100% (95% CI: 97.3–100% and 95.7–100%), with a concordance rate of 100% (95% CI: 98.4–100%) (Table 3).

**TABLE 3:** Sensitivity and specificity of phenotypic tests (95% CIs)

	CNPt	CNPt	BCT	c-CNPt
Sensitivity (%)	63.6 (56.0–70.6)	100 (97.3–100)	100 (97.3–100)	100 (97.3–100)
Specificity (%)	100 (95.7–100)	100 (95.7–100)	100 (95.7–100)	100 (95.7–100)
Accuracy (%)	77.4 (72.2–81.9)	100 (98.4–100)	100 (98.4–100)	100 (98.4–100)

## DISCUSSION

*Aeromonas*, significant opportunistic pathogens, are widely distributed across diverse ecological environments and can infect both humans and animals, positioning them as crucial indicators for monitoring antimicrobial resistance from a “One Health” perspective (19). Currently, 36 species of *Aeromonas* have been identified, with at least 19 capable of causing human infections, predominantly including *Aeromonas caviae* (37.26%), *Aeromonas dhakensis* (23.49%), *Aeromonas veronii* (21.54%), and *Aeromonas hydrophila* (13.07%) (20, 21). Studies have shown that both the VITEK2 and MALDI-TOF Biotyper systems are effective in accurately identifying *Aeromonas* at the genus level. However, they encounter significant challenges with species-level identification, leading to notable discrepancies (22, 23). In light of these limitations, we chose not to differentiate between species and instead concentrated our analysis at the genus level to minimize the risk of misidentification. The escalating emergence of antibiotic resistance in *Aeromonas*, particularly to carbapenems, has garnered widespread attention. Although carbapenem resistance in *Aeromonas* is primarily mediated by chromosomal *bla*CphA, plasmid-associated carbapenem resistance genes such as *bla*KPC, *bla*VIM, *bla*IMP, *bla*NDM, and *bla*OXA have also been identified. *Aeromonas* can serve as a reservoir for disseminating resistance genes among humans, animals, and the environment (24–26). Studies indicate that conventional AST often fails to fully detect carbapenem-resistant strains (6–8). Our prospective trial identified 97 isolates carrying carbapenem resistance genes, accounting for 63.8%. However, the resistance rate of four carbapenems in susceptibility tests ranged from 22.4% to 58.6%, potentially missing some CphA-positive strains, indicating that the VITEK2 system cannot be solely relied upon for accurate identification of carbapenemase-producing organisms. Consequently, early identification of carbapenem-resistant *Aeromonas* is essential for effective anti-infection treatment and antimicrobial management.

Currently, methods for detecting carbapenemase are primarily categorized into genotypic and phenotypic detection methods. Genotypic detection offers high specificity and sensitivity, but it is costly and complex to operate. Additionally, it cannot identify novel or unknown resistance genes, making it unsuitable for routine use in microbiology laboratories. Conversely, phenotypic detection is simpler to operate and cost-effective, making it suitable for preliminary screening in routine laboratories and resource-limited settings. Our previous research demonstrated that the mCIM could accurately detect carbapenemase in *Aeromonas* with 100% sensitivity and specificity (8). Despite its effectiveness, mCIM requires a longer processing time, hindering early detection. The CNPt, recommended by CLSI for *Enterobacterales* and *P. aeruginosa*, is another method for detecting carbapenemase. It is useful for treatment decisions, infection prevention, and epidemiological investigations, and it can yield results within 2 h. For *Enterobacterales*, the sensitivity is 84% and the specificity is 100% (27). For *P. aeruginosa*, both the sensitivity and specificity are 98% (27). Research indicates that in *Aeromonas*, CNPt detects CphA with a sensitivity of 97.4%, specificity of 95.7%, positive predictive value of 98.7%, and negative predictive value of 91.7% (7). However, our retrospective study found that out of 68 CphA-positive strains, only 38 tested positive for carbapenemase using CNPt. In a prospective study, of the 97 carbapenemase-producing strains, only 64 were CNPt positive. The sensitivity of CNPt for detecting carbapenemase in *Aeromonas* was thus only 63.6%, with all false-negative results occurring in CphA-positive strains. The speculated reasons for this discrepancy include (i) strain differences, where some *Aeromonas* strains exhibit low CphA enzyme expression, leading to inadequate imipenem hydrolysis and an insufficient H^+^ release for a visible color change; and (ii) methodological issues, as 100 µL extraction solution used in CLSI-CNPt may retain residual buffer, which can hinder the color shift, reducing detection sensitivity for carbapenemase-producing strains (16). Thus, CLSI-CNPt may not be ideal for detecting carbapenemase in *Aeromonas*.

Several improved CNPt methods, such as CNPd and BCT, offer streamlined procedures and cost-effective benefits, promising significant application potential. CNPd directly utilizes bacterial colonies for detection, circumventing buffer interferences, and incorporates Triton X-100 to enhance carbapenemase release, achieving 98% sensitivity and 100% specificity (16). Meanwhile, the BCT employs bromothymol blue as an indicator, covering an optimal pH range (6.0–7.6) for most lactamases (pH 6.8), demonstrating consistent 100% sensitivity and specificity (17). These methods reliably identify OXA-type carbapenemases and are suitable for rapid detection of carbapenemase production directly from blood cultures (16, 17, 28). The MBL CphA, located on the chromosome of *Aeromonas*, exhibits specific carbapenem hydrolysis activity but with modest efficiency. Conventional susceptibility tests do not adequately detect carbapenem resistance in the presence of CphA carbapenemase, and unmodified CNPt methods specifically fail to identify CphA-producing strains, potentially resulting in inappropriate carbapenem use and treatment failures. In our study, we applied CNPd and BCT for the first time to detect carbapenemases in *Aeromonas*, achieving 100% sensitivity and specificity in identifying CphA. These methods outperform conventional susceptibility tests, providing consistently accurate results that align with genetic testing.

Based on the aforementioned phenotypic tests, we designed a modified CNPt, named c-CNPt. This assay utilizes the same solutions A and B as the CLSI-CNPt but eliminates the need for protein extraction using a lysis buffer. By eliminating the protein extraction step, bacterial colonies are directly mixed with the reagent and incubated, allowing carbapenemase-producing *Aeromonas* strains to continuously express and secrete carbapenemase in response to imipenem. This accelerates imipenem hydrolysis and H^+^ release, resulting in a pH change and a clear color shift from red to yellow, signifying a positive result. This approach minimizes buffer interference and significantly improves the accuracy and reliability of detection. To further distinguish the types of carbapenemases, EDTA was incorporated into solution C of the ec-CNPt. This allows for the preliminary differentiation between serine carbapenemase and MBL, thereby providing crucial information for clinical therapeutic decision-making. The c-CNPt assay demonstrated a sensitivity and specificity of 100%, making it a rapid and precise method for identifying carbapenem-resistant *Aeromonas*. Additionally, we evaluated the stability of c-CNPt reagents and found that they could be stored at −80°C for at least 1 year without affecting the test results. Therefore, after initial preparation, the reagents can be aliquoted and stored in a −80°C freezer for convenient use, greatly facilitating routine testing in microbiology laboratories and making them highly suitable for regular applications.

This study has several limitations. First, although 131 clinical *Aeromonas* strains and 152 prospective isolates were included, the geographic and strain diversity may still be insufficient. Expanding the sample size to cover a wider geographical range could improve the robustness of the conclusions. Additionally, the traditional CNPt demonstrated a high rate of false negatives for CphA-positive strains, although improvements were seen with the modified tests. These tests, however, still require further optimization and validation across a broader range of bacterial species and resistance genes. Finally, although the tests employed in this study can detect carbapenemase activity, including chromosomally encoded CphA, they cannot differentiate between chromosomal and acquired carbapenemases (such as KPC and NDM). Therefore, further genotypic or more specific phenotypic assays are necessary for precise carbapenemase identification. Due to the limited number and variation of non-CphA carbapenemase producers evaluated, the ability of ec-CNPt to differentiate between NDM and KPC may not fully capture the variability observed in clinical settings. Further studies with a broader range of carbapenemase variants are necessary to validate these findings.

In conclusion, our research demonstrates that, despite certain limitations of the CLSI-CNPt method, the modified phenotypic assays, such as CNPd and BCT, exhibit superior sensitivity and specificity in detecting carbapenemase production in *Aeromonas*. The optimized c-CNPt assay does not require complex equipment or specialized personnel, thereby facilitating its application across various healthcare settings. Furthermore, the combination with ec-CNPt assay enables differentiation between different types of carbapenemases. Taken together, the c-CNPt and ec-CNPt assays offer significant advantages in carbapenemase detection, characterized by high sensitivity, specificity, rapid turnaround, and cost-efficiency. These attributes underscore their significant potential in managing antibiotic resistance and safeguarding public health. Nevertheless, further studies are needed to incorporate more diverse samples and carbapenemase types to validate the broad applicability of these methods.
